# An Overview of the Pathogenesis and Treatment of Elbow Osteoarthritis

**DOI:** 10.3390/jfmk4020030

**Published:** 2019-05-29

**Authors:** Silvia Ravalli, Carmelo Pulici, Stefano Binetti, Alessandra Aglieco, Michele Vecchio, Giuseppe Musumeci

**Affiliations:** 1 Department of Biomedical and Biotechnological Sciences, Human Anatomy and Histology Section, School of Medicine, University of Catania, Via S. Sofia n°87, 95124 Catania, Italy; 2 Department of Biomedical and Biotechnological Sciences, Division of Physical and Rehabilitative Medicine, University of Catania, via S. Sofia 67, 95123 Catania, Italy; 3 Research Center on Motor Activities (CRAM), University of Catania, via S. Sofia 97, 95123 Catania, Italy

**Keywords:** elbow arthritis, primary osteoarthritis, post-traumatic osteoarthritis, treatment, exercise

## Abstract

The elbow joint could be associated with degenerative processes of primary and post-traumatic aetiology. Among these, osteoarthritis may also be secondary to repeated use as well as trauma. Pain, discomfort and progressive loss of functionality are common signs of this condition. The evaluation of elbow osteoarthritis should comprise an in-depth study to detect the primary cause of the illness and to facilitate the decision-making process regarding personalized treatment. Discordance between clinical manifestations and radiological findings is common. Conservative approaches may provide symptomatic relief in the early stages of disease for most patients. The goal of the treatment is to reduce pain and ensure an adequate range of motion and proper functioning of the joint while preserving the anatomical structure, to postpone elbow arthroplasty interventions for as long as possible. According to treatment guidelines, surgery should be considered depending on aetiology and severity, patient age, and functional demands. This narrative review aims to investigate the current literature regarding the pathogenesis and treatment of primary and post-traumatic arthritis of the elbow.

## 1. Introduction

**Figure 1:**
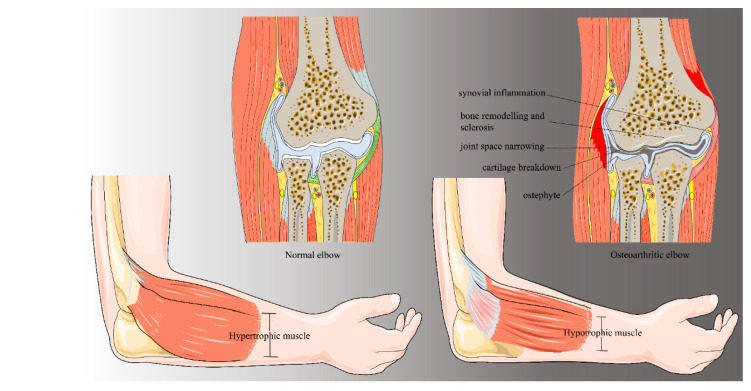
Differences between normal and osteoarthritic elbow.

Osteoarthritis (OA) is one of the most important diseases associated with ageing, being rated as the 4th leading cause of chronic disability, which is diagnosed in around 40% of elderly people (over 60 years of age). It is the most common cause of joint replacement in developed societies, and its financial and social burden is likely to increase because of extending longevity and increasing levels of obesity [1]. A study conducted in 2016 estimated that the average total cost, worldwide, per knee osteoarthritic patient, is 11.1 k€ per year [2]. OA is often driven by an abnormal or injurious mechanical loading on the joint, but the exact mechanism behind senile articular changes which contribute to the process remains unclear [3]. Several hypotheses have been proposed, but it is more likely that more than a single factor play a part in the increased risk of articular degeneration with ageing. These include (i) repetitive mechanical micro-trauma over several decades, (ii) reduced muscle strength, leading to loss of joint protection during normal use, (iii) reduced autophagy and increased cellular senescence, (iv) reduced reparative response of the articular cartilage to injury, and (v) loss of surface lubrication leading to increased friction and damage [4]. OA causes changes in mobility and functionality, and patients commonly experience daily physical limitations like difficulties while performing working tasks or problems with housekeeping [5]. Distinct biological processes are to be considered crucial for the development of OA, and they are assumed to act in concert with additional risk factors to promote the appearance of the symptoms. The leading cause is attributable to an imbalance between anabolic and catabolic processes within the cartilage tissue, resulting in loss of structure and functionality of the whole articulation (Figure 1). OA progresses when cartilage degradation exceeds self-reparative processes [6]. In addition to trauma, other causes include overuse injury, osteochondritis dissecans, osteochondromatosis, crystal-induced arthropathies, and the sequelae of septic arthritis or haemophilia [7]. The biomechanical environment experienced by the joint needs to be taken into account when considering weight-bearing as a possible risk factor. Even though the elbow is not subjected to the same weight pressure of the knee joint, the articular cartilage on the upper extremity could undergo similar tensile stresses moving from a flexed to an extended position [8]. When elbow arthritis is not asymptomatic, it may be recognized by pain and impaired ability of movement. Joint narrowing is more commonly found in other joints rather than the elbow, while osteophytes presence and capsular contracture, with or without loose bodies, are very frequent [9]. Functional elbow range of motion (ROM), required for normal daily living activities, is considered to be an angle of motion of 100° (30° of extension to 130° of flexion) and a 100° angle of forearm rotation (50° of pronation to 50° of supination) [10]. Early stages of elbow OA are commonly treated without requiring surgical procedures. Operative treatment options, ranging from arthroscopy to open surgery, are performed to improve ROM and provide relief from pain by removing osteophytes and releasing of contracted soft tissue [11]. This review aims to investigate the current literature regarding the pathogenesis of primary and post-traumatic arthritis of the elbow. The principles of prevention and non-operative management will be presented, as well as indications for operative treatments.

## 2. Background and Pathogenesis 

### 2.1. Primary Elbow Osteoarthritis

Less than 2% of the patients with elbow arthritis suffer from the rare condition of primary OA. The most at risk population is represented by men subjected to heavy daily movements that exhaust the arm, or to weight lifting [12]. Although OA has a multifactorial nature, genetic factors have been found to be strongly involved in the regulation of OA-related pathways like those controlling cartilage and bone turnover and remodeling, body weight, muscle mass, and injuries response [13]. Investigations have focused on genes which support the structural integrity of the cartilage extracellular matrix (COL2A1, COL9A3, COL11A1), genes which are involved in signal transduction pathways in chondrocytes (BMP5, FRZB, IL-4Rα) or in bone metabolism (Vitamin D Receptor), and genes linked to inflammatory cytokines (IL-1, TNFα) [14]. Candidate gene-based analyses and genome-wide association studies (GWAS) have made it possible to design valuable case-control studies on osteoarthritis. Furthermore, considerable progress has been made in recent years in analysing the function of epigenetic modifications and their interplay with polymorphisms [15]. Besides epigenetics, inflammation mechanisms are involved in the pathophysiology of OA. Articular damage could be triggered by a series of risk factors or conditions like age, obesity, injuries and, as already mentioned, genetics, evolving into a vicious cycle of local tissue breakdown and chronic low-grade inflammation which progressively leads to OA dysfunction. The inflammation results from damage-induced immune system activation. The presence of damage-associated molecular patterns (DAMPs) deriving from the breakdown of cartilage triggers chondrocytes, fibroblast-like synoviocytes and synovial macrophages to produce inflammatory key mediators: cytokines (e.g., TNF-α, IL1-β, IL-6), growth factors, chemokines, prostaglandins, leukotrienes, adipokines and others. Toll-like receptors (TLRs) and receptor for advanced glycation end products (RAGE) are the main pattern recognition receptors (PRRs) involved [16]. Detrimental changes are caused by matrix metalloproteinases (MMPs) and disintegrin and metalloproteinase with thrombospondin motifs (ADAMTSs). Failure to repair mechanisms results in amplified and unresolved inflammatory processes, hypertrophic chondrocyte, apoptosis, loss of homeostasis and, lastly, in clinical osteoarthritis [17]. The pattern of pain in patients with primary OA is classically characterized by the patient complaining of impingement pain when asked to extend or flex the elbow at the maximum range of movement, especially during extension. In addition, pain can be caused by the presence of osteophytes in the olecranon fossa and in the proximal portion of the olecranon, even if the intra-articular space is still preserved [18]. In a similar way, extreme flexion could be the cause of suffering for patients whose osteophytes sprout occurs in the trochlea or the coronoid process [10]. If the entire range of motion is associated with discomfort, it could be indicative of an advanced phase of the disease, since prolonged pain is generally a late symptom [19].

### 2.2. Post-Traumatic Osteoarthritis

Post-traumatic OA may occur in the elbow as a result of traumatic injury. Men and women of all ages could be affected by this form of OA, although it is most common in young males [20]. The risk of developing this pathology depends on injury extent, gravity and on the way the trauma is provoked. Intra-articular distal humerus fractures, for example, are frequently associated with degenerative joint complications over time [21]. Joint kinetics could be altered by ligament instability, which can also participate in the worsening of pre-existing conditions [22]. Serious injuries can also cause capitellar osteochondritis dissecans (OCD), especially in young athletes, affecting the entire articular cartilage structure [8]. Patients who have post-traumatic arthritis experience pain throughout the entire arc of motion [19].

### 2.3. Elbow Osteoarthritis in the Athlete

The elbow undergoes important mechanical stress during physical activity [23]. Injuries occur mostly in “overhead athletes”, i.e., those who move their upper arm and shoulder over their head during performance. Throwing or swinging are critical movements, so volleyball, golf, tennis and baseball players are most commonly affected by this kind of trauma. The elbow might also be damaged in contact sports such as rugby and martial arts, and because of falls occurring during sports like gymnastics. An athlete can unwittingly force the kinetics of his elbow beyond the point of rupture and impose additional stress, leading to injury, altered mechanics, and ultimately OA. OA of the elbow in athletes may be secondary to repetitive use, as well as being linked to trauma both in adults and adolescents; in the latter case, as mentioned above, osteochondritis desiccants of the capitellum may develop as well [24]. Secondary changes, such as the formation of osteophytes in the olecranon and coronoid process, loose bodies, and chondromalacia of the radius and capitellum, are common, and may occur at a relatively young age, particularly in athletes involved in throwing objects, such as shot put or javelin throw athletes [24]. Long-term, osteoarthritic signs are seen in many athletes, and can lead to a decreased range of motion and pain throughout the range of motion [25]. Careful patient history recording and thorough examinations are paramount for reliable diagnosis; in fact, other kinds of injury or dislocations must be considered as well. OCD, for example, should be considered in athletes whose elbows are subjected to chronic shear stress, like basketball players, and can incur microtrauma or ischemic events [26]. Players who experience pain in extension could also suffer from olecranon traction spur or synovial impingement [27]; difficulties during the rotation of the arm may indicate radioulnar synostosis [28]. 

## 3. Clinical and Radiological Evaluation 

### 3.1. Patient Evaluation 

Aetiological investigation of elbow osteoarthritis starts with an accurate recording of the patient’s history. It is more likely that patients who are less than forty years old experience pathological condition as a consequence of a previous trauma [11]. It is essential to be aware if the patient performs a daily working routine which requires strenuous manual labor, since it can influence the degree of pain and disability. Other important aspects that should be investigated are the extent and location of the symptoms and their absence or worsening at rest/during the night. In cases of primary OA, evaluation of radiocapitellar joint needs to take into account that its degeneration is not always accompanied by pain, except during forearm rotation [29,30]. Visual examination gives information about the external integrity of the elbow. The lateral epicondyle, the olecranon, and the radial head together form a posterolateral soft spot that should be palpated to detect effusions. Movements of flexion-extension and pronation-supination are performed to evaluate disability, pain sensibility and crepitus as typical signs of arthritis presence. While extensive lesions are a source of pain during the mid-range of motion, osteophyte impingement is commonly associated with pain during forced movements. Neurovascular integrity should be checked as well, paying particular attention to the condition of the ulnar nerve. Planning a surgical treatment needs to take into account previous procedures involving repositions of this nerve. Also, any knowledge of prior surgery may indicate infection. In this case, synovial fluid analysis is strongly recommended, as well as counts of blood-cell, erythrocyte sedimentation rate and C-reactive protein levels [11]. 

### 3.2. Rating Systems for Evaluation of the Elbow

Many scoring systems have been used to rate functional aspects in elbow disorders. However, only a few of these have been validated. Although many implementations have been made, a gold standard evaluation which is practical, not time-consuming and accurate enough to detect clinically relevant worsening or improvements does not exist yet [31]. A good score system is a fundamental tool for establishing the seriousness of disability, keeping track of the progresses and comparing treatments. Observer-based approaches and patient-completed functional questionnaires are the two main types of scoring evaluation that are broadly used [32,33,34]. The Mayo Elbow Performance Score (MEPS) analyses four categories: pain, range of motion, stability and daily function. Each category has a series of questions whose answers are marked with a score. The sum of all the scores can be correlated with an index of functionality ranging from excellent to poor. Turchin et al. compared different observer-based systems in order to establish their validity [35]. From the examination, it was possible to state that, while there is a good concordance between raw scores, the correlation between categorical ranking, of different systems, is low. The patient-completed functional questionnaire is, instead, a subjective method of evaluation, since it does not comprise physical check and external observation.

Regarding this approach, Turchin et al. suggest that sometimes these models perform even better than the observer-based ones [35]. Nonetheless, critical clinical features, useful to surgeons, are not directly measured by the questionnaire. The Disabilities of the Arm, Shoulder and Hand (DASH) questionnaire is one of the most common [36]. The Research Committee of the American Shoulder and Elbow Surgeons (ASES) created a scoring system which merges both a self-reporting and an observational judgment [37]. Personal experience of pain has a strong influence on each criterion of assessment and can distort objective evaluations [11,38]. Psychological and sociological circumstances also play a role in pain manifestation: mental health conditions, like depression, anxiety, schizophrenia, bipolar disorders and others contribute to patient perceptions of clinical state and understanding of medical circumstances, influencing the overall experience of discomfort. As a consequence, it is necessary to adopt pain management strategies which look at these sensitive aspects in terms of vulnerabilities and strengths [39]. In addition, socio-economic status influences the progression of osteoarthritic disease and the management of pain, since high grades of poverty and low levels of education are more frequently associated to labor-intensive occupations (which can lead to trauma and disability), minor awareness about environmental risk factors or daily precautionary measures, difficulty to access to healthcare system and high percentage of comorbidity [40]. Underestimation of physical improvements, especially following surgical procedures, is commonly due to low tolerance for pain. Therefore, it would be useful to exclude this information from objective evaluations, avoiding distortions of the real outcomes of surgery [31].

### 3.3. Imaging

**Figure 2:**
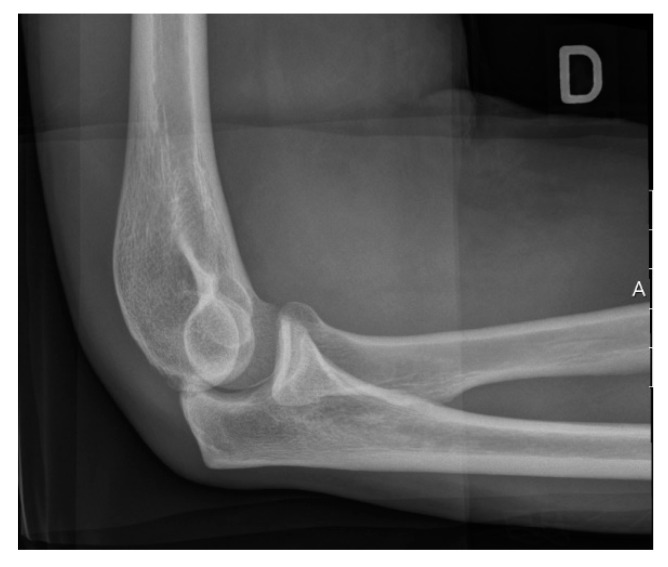
Lateral radiograph of the elbow in a healthy patient.

**Figure 3:**
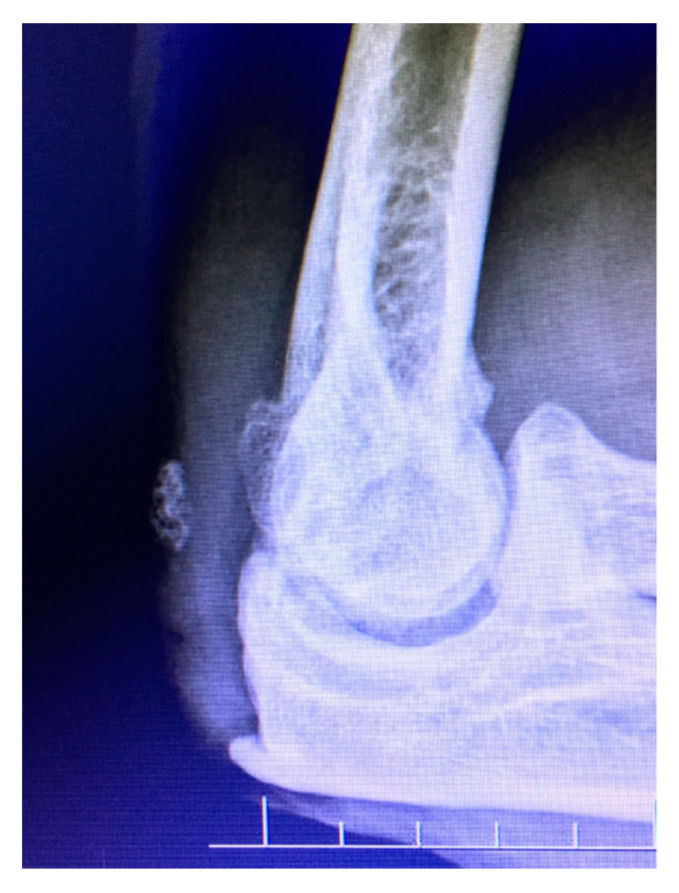
Lateral radiograph demonstrates idiopathic arthritis with osteophytes on the radial head, as well as anterior and posterior ulno-humeral joints.

Anteroposterior and lateral plain radiographs are usually sufficient in initial assessments of elbow arthritis [41] (Figure 2 and Figure 3). Primary osteoarthritis is radiographically detected by noticing characteristic osteophytes on the coronoid process and olecranon. The absence of hypertrophic osteophytes, but the presence of severe joint space narrowing typically suggest inflammatory arthritis. Loose bodies are not easily detected on radiographs and could be missed [42,43]. Further imaging examination can be necessary to visualize loose bodies which are present in the posterior compartment and proximal radioulnar joint [44]. The use of computed tomography or magnetic resonance arthrography is recommended, as these tools can aid in detecting and locating loose bodies and/or impinging osteophytes [11].

## 4. Treatment 

### 4.1. Conservative Treatment

Treatment aims to reduce pain and improve articular function and global stability. During the early stages of elbow OA, physicians try to arrest or alleviate pain by instructing the patient on which type of movements need to be avoided, by prescribing nonsteroidal anti-inflammatory drugs (NSAIDs) such as aspirin and ibuprofen, and/or by acting on-site through intra-articular glucocorticoid injections and visco-supplementation. The latter has shown to exert limited effects in the elbow joint and still requires further studies to ascertain if it is genuinely beneficial and can provide long-term benefits [7]. To give an example, a study involving patients with post-traumatic OA showed that pain relief after visco-supplementation lasted for six months at the most [45]. Rehabilitation is a fundamental tool to slow down OA elbow deterioration. The primary goal of therapy should be to properly inform the patient about his/her symptoms and how to try to control them, reducing the pain and preserving the wellness of the elbow. However, beneficial changes in routine activities can be problematic for physically active athletes [27]. In fact, in order to limit the pain and reduce inflammation, the patient could be asked to reduce movements. An elbow splint can be suggested to immobilize and protect the elbow [11]. Range-of-motion (ROM) and stretching exercises should be started at the earliest pathological signs. These exercises can improve elbow flexibility, increase stability and protect the joint from mechanical stress [46]. Rehabilitation processes need to involve specific exercises also for hip stability, core muscles and scapula, shoulder and hand. The patient will follow-up with a home program within four to six weeks [47]. A joint protection plan is thought to help the patient to perform activities of daily living (ADLs), aiming to control pain, minimize further joint deterioration, and preserve energy [48]. It is also essential to prevent non-physiological positions and maintain balance during weight lifting. Night splints could also help to protect the elbow during sleeping hours [19]. The primary purpose of treatment, especially in young patients, is to achieve minimal pain and to retain proper articular functioning while preserving the joint from future surgical interventions and delaying arthroplasty for as long as possible [49].

### 4.2. Surgical Treatment

**Figure 4:**
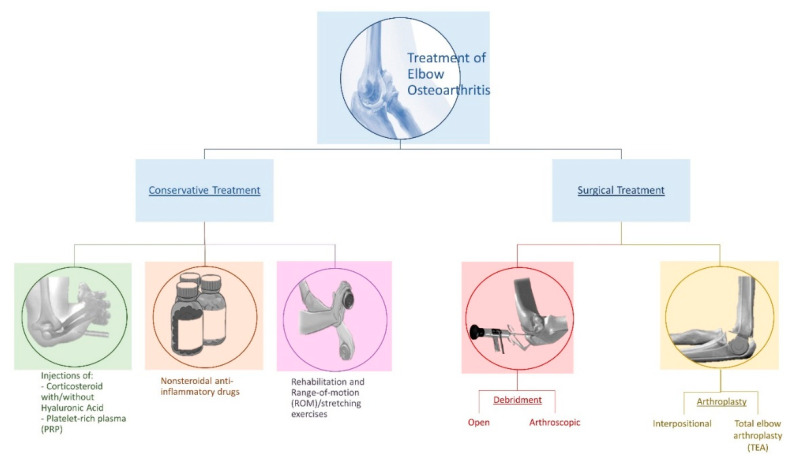
Main treatment approaches for elbow osteoarthritis.

Surgery represents the best option if the symptoms are at an advanced stage. Several factors, such as severity of degeneration, age of the patient and level of physical activity, need to be taken into account, considering which kind of procedure is most suitable for each patient [50]. Any surgical procedure, ranging from open surgery to mini-invasive arthroscopy, aspire to remove osteophytes and release the contracted soft tissue in order to ameliorate the range of motion and provide relief from pain [27]. The surgical procedures include (1) diagnostic biopsies, (2) disease debulking synovectomy, (3) regaining of motion and capsulectomy, and (4) pain relief, with or without radial head excision and joint replacement [50]. Open debridement of the elbow has favorable outcomes in terms of the improved range of motion, and long-term results seem to be reliable and consistent. Infection is one possible risks, as well as injury in proximity of the neurovascular structure, stiffness and thrombosis. The presence of persistent ulnar neuropathy could negatively influence the outcome of this technique and, in many cases, like in young athletes, it would be more appealing to opt for a less invasive approach like arthroscopy [27]. Elbow arthroscopy offers the advantage of decreasing post-operative pain, reducing arthrofibrosis, facilitating rehabilitation and, as a consequence, recovery, especially for athletes who practice their sport on a daily basis [51]. Cohen et al. [52] compared open and arthroscopic debridement, reporting better results for the latter procedure. Despite being a generally safe procedure, arthroscopy could present some risks associated with open procedures like infection and heterotopic ossification. Other complications could be the presence of fistulae or the necessity of persistent drainage and neurovascular damage. The latter is quite difficult to avoid and commonly involves the ulnar nerve, followed by the superficial radial nerve, posterior interosseous nerve, anterior interosseous nerve, and medial antebrachial cutaneous nerve [53]. Interpositional arthroplasty represents an alternative to total elbow arthroplasty (TEA) in young adults [54]. This approach uses autograft material (fascia lata, cutis) or allograft material (e.g., Achilles tendon, dermis) to resurface the elbow articular surface, mainly by introducing a cushion of thick and hard tissue between the bones [49]. It works reasonably well in the elbow, but is also complicated by the standard risks deriving from the operative intervention, and the possible development of post-operative inflammatory state, weakness or paresthesia involving nerves [54]. However, TEA remains the definitive functional procedure for end-stage OA, despite not being a viable option for active young patients [27,49]. TEA is used for patients with significant loss of elbow function and severe elbow pain and stiffness. The goal of this approach is to maximize the longevity of the implant [50]. Complications associated with TEA are infections sustained by Methicillin-resistant Staphylococcus aureus (MRSA) or Staphylococcus epidermidis; heterotrophic ossification; ulnar neuropathy; articular instability and, most frequently, aseptic loosening [55]. It is also good to remember that, even after successful elbow arthroplasty, the patient has to limit lifting in terms of weight and repetition. Padding bandages and elbow splints will serve as supports after surgery, and physiotherapy sessions are recommended for at least the following three months. Rehabilitation therapy will serve, at first, to limit the physiologic pain and swelling caused by the surgery, and then, to reinforce and stabilize the muscles around the joint [11] (Figure 4).

### 4.3. Alternative and New Approaches

Young patients and athletes could benefit most from alternative or non-invasive treatments, and early cartilage damage could recover without involvement of important surgical techniques. As already mentioned, injections of corticosteroids in association with hyaluronic acid, which has lubricant and shock absorber properties, represent a simple approach with minimal side effects, but offer limited and short-term improvements. Injections of platelet-rich plasma (PRP) seem to overcome steroid injections in term of outcomes and side effects. PRP comprises platelets and growth factors deriving from centrifugation of whole blood. This mixture is used in elbow pathologies in order to help regenerating soft tissue through releasing the cytokines which are able to modulate inflammatory responses [56]. Another opportunity to reduce pain intensity could be offered by denervation techniques, which have been shown to be effective at the wrist [39]. Even though total denervation of the elbow appears impossible, partial denervation should be able to maintain mobility while managing pain [57]. Among surgical procedures, novelty in arthroplasty relies on newer implant designs and materials in order to meet mechanical burden and anatomic reconstruction. In addition, press-fitted components and cementless options are suggested to avoid risk of aseptic loosening [58]. Small lesions could also be treated by osteochondral autograft transplantation, providing an opportunity to preserve hyaline cartilage, restore joint congruity and function and reducing risk of damage progression. Lastly, a promising application in regenerative medicine is represented by the use mesenchymal stem cells (MSCs) to restore damaged articular cartilage. Biocompatible scaffolds and bioreactors, used to expose the cells to shear and compression loadings, are currently the object of research [59].

## 5. Conclusions

Our work has allowed us to detect improved prevention of OA, which remains a significant obstacle to physical rehabilitation. Avoiding elbow injury, muscular weakness and imbalance are precautionary measures to reduce risk factors. Osteoarthritis of the elbow is not a common condition, but it is associated with severe pain and disability, especially in athletes. Sport activities subject the joint to supra-physiological stress, which will deteriorate the articulation in the long run. Weak muscles, in addition, impede motor control and reflexes. If muscles lose their protective function, the joint will lack stability. As a consequence, microtrauma on the articular cartilage is more likely to occur, paving the way to degeneration processes which characterize OA. Exercises that increase muscle mass and global function could be considered crucial in the prevention of OA [60]. Patients, physicians, coaches and athletes should remain vigilant for the signs of muscle tiredness, including decreased speed ability or accuracy during movements. Two to three successive months of rest (four months is preferable) from any throwing activities should occur each year [61]. An accurate history of the patient’s symptoms and clinical signs should be carefully assessed. An attentive functional analysis of the elbow joint is essential to perform an early diagnosis and to avoid a worsening clinical condition. Advanced imaging and functional assessments can confirm the diagnosis and help in deciding between non-surgical treatments or surgical intervention. The latter must be considered when conservative treatment fails to provide symptomatic relief. There are several surgical options available for the physician. Elbow arthroscopic debridement for primary degenerative osteoarthritis results in statistically significant and clinically relevant improvements in elbow ROM, low risks of complications, and reduced need for further surgical interventions.
